# Timeliness of antiretroviral therapy initiation in the era before universal treatment

**DOI:** 10.1038/s41598-021-90043-7

**Published:** 2021-05-18

**Authors:** Nikolina Bogdanić, Liam Bendig, Davorka Lukas, Šime Zekan, Josip Begovac

**Affiliations:** 1 University Hospital for Infectious Diseases, Mirogojska 8, Zagreb, Croatia; 2 Medical Scholars Program, AU/UGA Medical Partnership, Athens, GA USA; 3 University of Zagreb School of Medicine, Zagreb, Croatia

**Keywords:** Microbiology, Diseases, Health care

## Abstract

We assessed the prevalence and factors related to the time to antiretroviral (ART) initiation among persons who entered HIV care and subsequently started ART in Croatia from 2005 to 2014. Included were patients ≥ 18 years, the follow-up ended on Dec/31/2017. 628 patients were included into the study 91.9% were men; median age was 36.1 (Q1–Q3: 29.6–43.8) years. Rapid (within 7 days of diagnosis) ART initiation was observed in 21.8% patients, 49.8% initiated ART within 30 days, 21.7% and 28.5% had intermediate (31 days–1 year) and late initiation (> 1 year), respectively. Of 608 patients that achieved an undetectable viral load, 94% had a plasma HIV-1 RNA < 50 copies/ml at last measurement after a median follow-up of 5.2 years. On quantile regression analysis, calendar year of entry into care, and markers of more advanced HIV disease (higher viral load, lower CD4 cell count and clinical AIDS) were significantly associated with earlier ART initiation. Early ART was not related to a gap in care afterwards at all quantiles. In conclusion, a significant proportion of patients started ART early in Croatia in 2005–2014. Early ART initiation led to durable viral load suppression and was not associated with a subsequent gap in care.

## Introduction

Guidelines concerning antiretroviral therapy (ART) initiation have evolved over the past decade. While there was a consensus that ART should be started as soon as possible in patients with clinical AIDS, a CD4 cell count threshold for ART initiation has been debated since the introduction of modern ART in 1996. In 2010 WHO recommended ART for patients with a CD4 cell count beneath 350 cells/µL^1^. The landmark START trial^2^ in conjunction with other randomized trials^3^ and observational studies^4,5^ led to a policy change in 2015 by recommending ART for all patients regardless of the CD4 cell count^6^. Another WHO update in 2017 recommended rapid initiation (within 7 days of diagnosis) of ART to indicated, willing patients^7^. These guidelines also raised concerns over the elimination of the series of standard counseling sessions preceding ART due to a possibility of resulting physical or psychological harm and potential non-adherence^7^. The 2020 European AIDS Clinical Society guidelines also mention the importance of rapid ART initiation but emphasizes patient understanding and readiness to begin, as do current U.S. Department of Health and Human Services recommendations^8,9^.

Several low- and middle-income countries have introduced rapid ART initiation as a means to address the challenge of follow-up loss and late patient presentation to care^10^. This policy is linked to faster viral suppression for people living with HIV (PLWH) which also means that the risk of onward transmission can be greatly reduced^10^. Another consideration in rapid ART is the feasibility of implementation. Retrospective evaluation of rapid-start ART in community settings at risk for loss to follow-up, comorbidities, and other vulnerabilities show that implementation is possible and successful in these instances^11,12^. Even in the setting of high HIV prevalence communities experiencing a variety of health care challenges, the goal of reducing time to ART initiation from diagnosis is achievable^11^. However, a recent study from the French Dat’AIDS cohort examining patients diagnosed with HIV between 2010 and 2015 reported that a longer time between first encounter and first ART prescription was associated with a better 1-year retention and higher viral load suppression at 6 months^13^.

In the era before universal ART, data on the timing of ART initiation from a patient’s documented HIV infection or first clinical visit was generally not highlighted and seldom reported. The aim of our study was to examine the timeliness of ART initiation among persons who entered HIV care in Croatia from 2005 to 2014. We sought to identify factors associated with differences in timing of ART.

## Results

A total of 628 patients were included into the study with a total of 4333.0 years of follow-up, of which 3684.9 (85%) years of follow-up were after ART initiation. The median follow-up time per person was 6.7 (Q1–Q3: 4.3–9.5) years and the median time on ART was 5.4 (Q1–Q3: 3.5–8.2) years. The main characteristics of our study population are presented in Table 1. There was a trend for earlier initiation of ART among older patients, those with more severe HIV-disease (lower CD4 cell count, clinical AIDS, higher viral load). Earlier ART initiation was observed more frequently in the more recent period (2012 to 2014) and among those living outside Zagreb as well as among persons with heterosexual mode of transmission (Tables 1 and 2). Among 244 patients with a CD4 cell count ≥ 350/mm^3^ at inclusion into care ART initiation within 30 days of HIV diagnosis was observed in 40 (16.4%), it was more frequent in the period 2012 to 2014 (30/101, 29.7%) compared to the period 2005 to 2011 (10/143, 7.0%). Median time from confirmed HIV test to first clinical visit was 7 (Q1–Q3: 2–15) days; this integration into care was within 7 days in 339 (54.0%) and within 1-month in 540 (86.0%) patients.

**Table 1:** Main characteristics of the entire study cohort and of each group based on antiretroviral therapy initiation after confirmed HIV diagnosis.

Variables	Total N = 628 (%)	ART initiation after confirmed HIV diagnosis				P-value
		Rapid ≤ 7 days N = 137 (%)	Early 8–30 days N = 176 (%)	Intermediate 31 day–1 year N = 136 (%)	Delayed > 1 year N = 179 (%)	
**Baseline characteristics**						
Male sex	577 (91.9)	122 (89.1)	163 (92.6)	126 (92.6)	166 (92.7)	0.297
Age, years	36.1 (29.6–43.8)	39.3 (32.8–48.0)	38.1 (30.9–46.8)	34.3 (27.8–43.1)	32.7 (28.2–39.1)	< 0.001
Age groups						< .001
< 30 years	169 (26.9)	21 (15.3)	39 (22.2)	44 (32.4)	65 (36.3)	
30–50 years	374 (59.6)	90 (65.7)	103 (58.5)	77 (56.6)	104 (58.1)	
> 50 years	85 (13.5)	26 (19.0)	34 (19.3)	15 (11.0)	10 (5.6)	
Mode of transmission						0.050^a^
MSM	476 (75.8)	99 (72.3)	129 (73.3)	106 (77.9)	142 (79.3)	
Heterosexual contact	121 (19.3)	31 (22.6)	40 (22.7)	22 (16.2)	28 (15.6)	
IDU	11 (1.8)	2 (1.5)	1 (0.6)	4 (2.9)	4 (2.2)	
Unknown	20 (3.2)	5 (3.6)	6 (3.4)	4 (2.9)	5 (2.8)	
Periods of integration into care, years						< 0.001
2005–2008	220 (35.0)	46 (33.6)	40 (22.7)	44 (32.4)	90 (50.3)	
2009–2011	176 (28.0)	38 (27.7)	51 (29.0)	37 (27.2)	50 (27.9)	
2012–2014	232 (36.9)	53 (38.7)	85 (48.3)	55 (40.4)	39 (21.8)	
Residency in Zagreb	273 (43.5)	61 (44.5)	71 (40.3)	41 (30.1)	100 (55.9)	0.074
CD4 count at inclusion into care, cells/μL	269.0 (69.0–445.0)	93.0 (34.0–209.0)	107.0 (31.5–269.5)	316.0 (169.0–456.0)	484.0 (372.0–633.0)	< 0.001
CD4 count groups at inclusion into care, cells/μL						< .001
< 100	190 (30.3)	71 (51.8)	87 (49.4)	24 (17.6)	8 (4.5)	
100–350	194 (30.9)	50 (36.5)	65 (36.9)	53 (39.0)	26 (14.5)	
≥ 350	244 (38.9)	16 (11.7)	24 (13.6)	59 (43.4)	145 (81.0)	
Viral load at inclusion into care, log10	5.1 (4.5–5.7)	5.6 (5.1–6.1)	5.4 (4.9–5.8)	5.1 (4.4–5.5)	4.5 (4.0–5.0)	< .001
Viral load at inclusion into care > 100 000 c/mL	341 (54.3)	105 (76.6)	117 (66.5)	74 (54.4)	45 (25.1)	< .001
Hepatitis B antigen present at inclusion into care	34 (5.4)	5 (3.6)	11 (6.3)	7 (5.1)	11 (6.1)	0.462
Hepatitis C antibody positive at inclusion into care	19 (3.0)	1 (0.7)	6 (3.4)	6 (4.4)	6 (3.4)	0.197
AIDS at inclusion into care present	186 (29.6)	49 (35.8)	81 (46.0)	29 (21.3)	27 (15.1)	< 0.001
**Follow-up characteristics**						
CD4 count at ART initiation, cells/μL	225.5 (62.0–355.5)	93.0 (34.0–209.0)	107.0 (31.5–273.0)	265.5 (154.5–401.0)	330.0 (229.0–454.0)	< .001
CD4 count groups on ART, cells/μL						< 0.001
< 100	211 (33.6)	71 (51.8)	87 (49.4)	25 (18.4)	28 (15.6)	
100–350	253 (40.3)	51 (37.2)	63 (35.8)	69 (50.7)	70 (39.1)	
≥ 350	164 (26.1)	15 (10.9)	26 (14.8)	42 (30.9)	81 (45.3)	
Viral load at ART initiation, log10	5.2 (4.6–5.7)	5.6 (5.1–6.1)	5.4 (4.9–5.8)	5.1 (4.5–5.5)	4.6 (4.3–5.3)	< .001
ART type						0.003^b^
2NRT + NNRTI	424 (67.5)	77 (56.2)	124 (70.5)	99 (72.8)	124 (69.3)	
2NRTI + PI	170 (27.1)	55 (40.1)	42 (23.9)	34 (25.0)	39 (21.8)	
2NRTI + II	26 (4.1)	2 (1.5)	7 (4.0)	2 (1.5)	15 (8.4)	
Other	8 (1.3)	3 (2.2)	3 (1.7)	1 (0.7)	1 (0.6)	
Had gap in care	120 (19.1)	15 (10.9)	14 (8.0)	21 (15.4)	70 (39.1)	< 0.001
Died	50 (8.0)	10 (7.3)	21 (11.9)	8 (5.9)	11 (6.1)	0.267

Values are frequencies, percentages and median with first and third quartiles (Q1, Q3).

*MSM* men who have sex with men; *IDU* injecting drug use; *ART* antiretroviral therapy; *NRTI* nucleoside analogues reverse transcriptase inhibitors; *NNRTI* nonnucleoside reverse transcriptase inhibitors; *PI* protease inhibitors, *II* integrase inhibitors.

^a^Cohrane-Armitage trend test for comparison of MSM *versus* heterosexual mode of transmission.

^b^Cohrane-Armitage trend test for the comparison of 2NRTI + NNRTI *versus* 2NRTI + PI.

**Table 2:** Time from HIV diagnosis to antiretroviral treatment initiation and to viral suppression (< 50 copies/ml) in 628 individuals entering care in Croatia from 2005 to 2014.

Variables	N (%)	Time from HIV diagnosis to ART initiation, months	Time from HIV diagnosis to viral suppression (< 50 c/mL), months
**Gender**			
Male	577 (91.9)	1.0 (0.8, 1.4)	9.8 (8.1, 10.9)
Female	51 (8.1)	0.6 (0.3, 1.9)	5.9 (4.3, 8.3)
**Place of residence**			
Zagreb	273 (43.5)	1.2 (0.7, 3.3)	12.5 (10.1, 15.1)
Other than Zagreb	355 (56.5)	1.0 (0.7, 1.3)	7.7 (6.7, 9.0)
**Mode of transmission**			
MSM	476 (75.8)	1.1 (0.8, 1.9)	10.1 (8.3, 11.6)
IDU	11 (1.7)	3.4 (0.1, 22.1)	9.9 (4.1, 35.7)
Heterosexual contact	121 (19.3)	0.6 (0.5, 1.0)	6.8 (5.8, 8.9)
Unknown	20 (3.2)	0.8 (0.2, 7.3)	6.5 (3.7, 21.0)
**Had AIDS**			
Yes	186 (29.6)	0.6 (0.5, 0.7)	6.5 (5.8, 8.3)
No	442 (70.4)	2.6 (1.3, 3.8)	11.1 (9.1, 13.2)
**Periods of entry into care, years**			
2005–2008	220 (35.0)	2.7 (1.3, 8.6)	11.2 (8.6, 16.2)
2009–2011	176 (28.0)	1.0 (0.7, 2.1)	8.4 (6.7, 11.6)
2012–2014	232 (36.9)	0.6 (0.5, 0.7)	8.1 (6.8, 10.1)
**Hepatitis B antigen present at inclusion into care**			
Yes	34 (5.4)	1.1 (0.5, 7.3)	7.3 (4.2, 27.3)
No	594 (94.6)	1.0 (0.7, 1.3)	9.7 (8.0, 10.6)
**Hepatitis C antibody positive at inclusion into care**			
Yes	19 (3.0)	2.4 (0.7, 13.7)	9.6 (4.5, 58.5)
No	609 (97.0)	1.0 (0.7, 1.3)	9.3 (7.9, 10.6)
**CD4 count at inclusion into care cells/μL**			
< 100	190	0.4 (0.3, 0.5)	6.2 (5.8, 6.5)
100–350	194	0.5 (0.5, 0.7)	6.4 (5.9, 7.2)
≥ 350	244	18.5 (14.1, 23.2)	26.7 (21.2, 31.1)
**Gap in care**			
Yes	120	26.0 (8.9, 35.5)	38.2 (26.9, 49.9)
No	508	0.7 (0.6, 0.9)	7.5 (6.8, 8.7)
**Integration into care, days**			
0–7	339 (54.0)	0.4 (0.3, 0.5)	6.5 (6.1, 7.0)
8–29	201 (32.0)	3.4 (1.2, 7.6)	12.1 (9.8, 15.1)
≥ 30	88 (14.0)	11.6 (3.7, 18.5)	19.2 (12.3, 30.0)
**VL > 100 000 copies/ml at inclusion into care**			
Yes	341 (54.3)	0.5 (0.4, 0.7)	7.4 (6.6, 8.6)
No	287 (45.7)	8.6 (3.8, 14.9)	16.1 (10.3, 21.1)

Values are frequencies, percentages, medians and 95% confidence intervals (CI). Medians and 95% CI are derived from the product-limit (Kaplan–Meier) estimates.

*MSM* men who have sex with men, *IDU* injection drug users, VL, NRTI, nucleoside analogues reverse transcriptase inhibitors, *NNRTI* nonnucleoside reverse transcriptase inhibitors, *II* integrase inhibitors, *PI* protease inhibitors, *VL* viral load.

### Timeliness of ART

**Figure 1:**
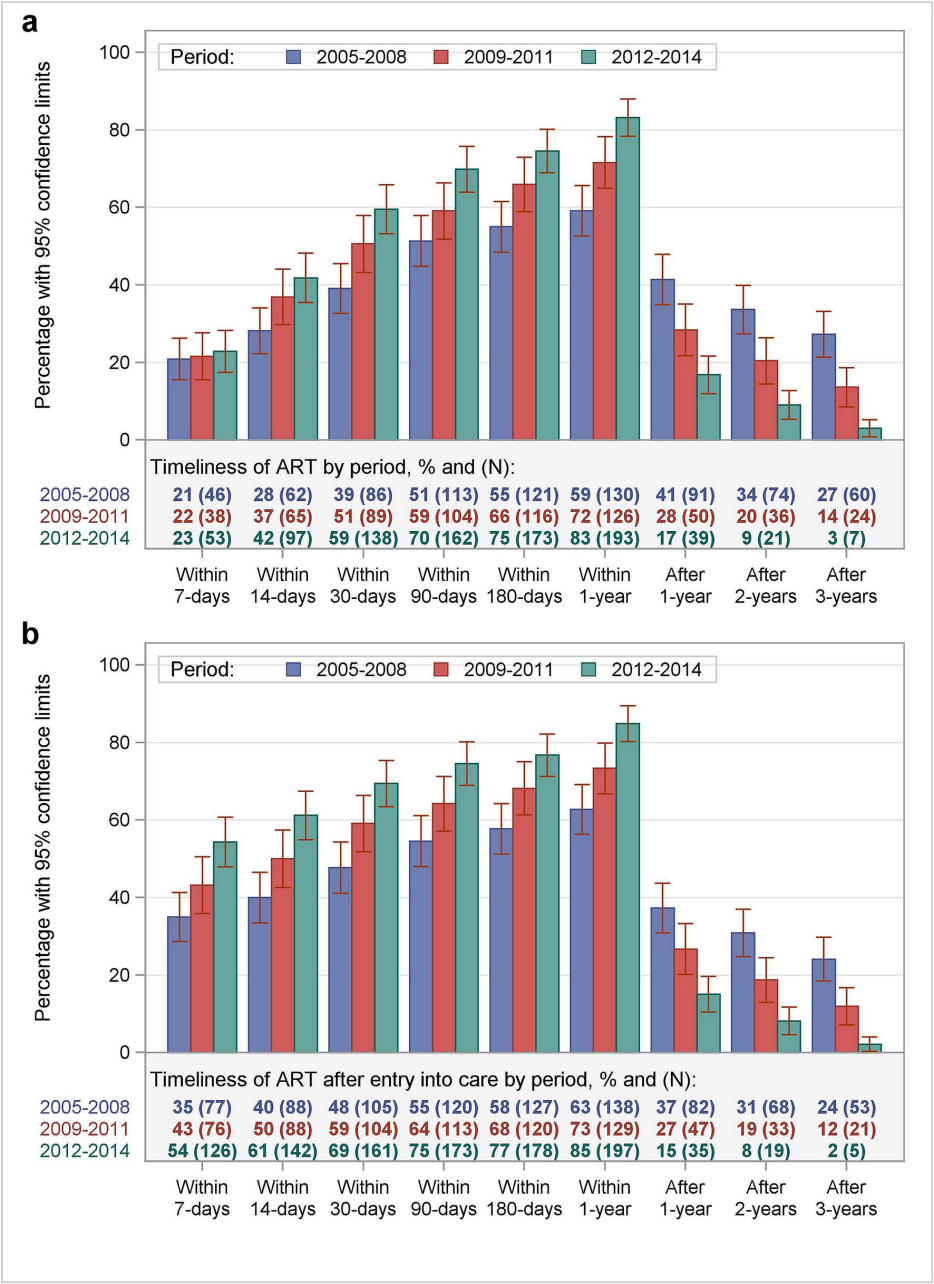
Time from HIV diagnosis to antiretroviral therapy (ART) initiation according to study periods. (**a**) Shows the frequency distribution of time from HIV diagnosis to ART initiation and (**b**) shows the frequency distribution of time from entry into care to ART initiation.

The median time from HIV diagnosis to ART initiation was 31 days (Q1–Q3: 8–537.5 days; 0.3–17.7 months), decreasing by 77.8% from the period 2005–2008 to 2012–2014 (Table 2). The proportion of patients with early ART (< 30 days of confirmed diagnosis) was significantly greater in the period of 2012–2014 than from 2005 to 2008 (Fig. 1a). About 20% of patients started ART within 7 days of HIV-diagnosis, but there was no statistically significant difference in different study periods (Fig. 1a, P = 0.618). The proportion of patients entering care from 2005 to 2008 also had significantly greater delay in ART initiation of more than one, two or three years after diagnosis (Fig. 1a).

In patients entering care between 2005 and 2008, the median time from the first clinical visit to ART initiation was 1.3 months (41 days) (Q1–Q3: 0.1–34.8 months; 4–1058 days). Patients from 2009 to 2011 had a median time from HIV diagnosis to ART initiation of 1.0 months (Q1–Q3: 0.3–16.9 months) and 0.5 months (14.5 days) from first clinical visit to ART initiation (Q1–Q3: 0.0–13.6 months; 1–412.5 days). Patients in the most recent studied time period, 2012–2014, had the earliest initiation of ART, the median time from diagnosis to ART initiation was 0.6 months (18 days) (Q1–Q3: 0.3–6.2 months; 8–188 days) and 0.2 months (6 days) (Q1–Q3: 0.0–3.3 months; 1–99.5 days) from the first clinical visit.

The proportion of patients starting ART from the date of first clinical visit follows the pattern of the time from HIV diagnosis to ART initiation (Fig. 1b). Median times to ART initiation and 95% CI for different patients’ characteristics are presented in Table 2.

### Viral suppression

**Figure 2:**
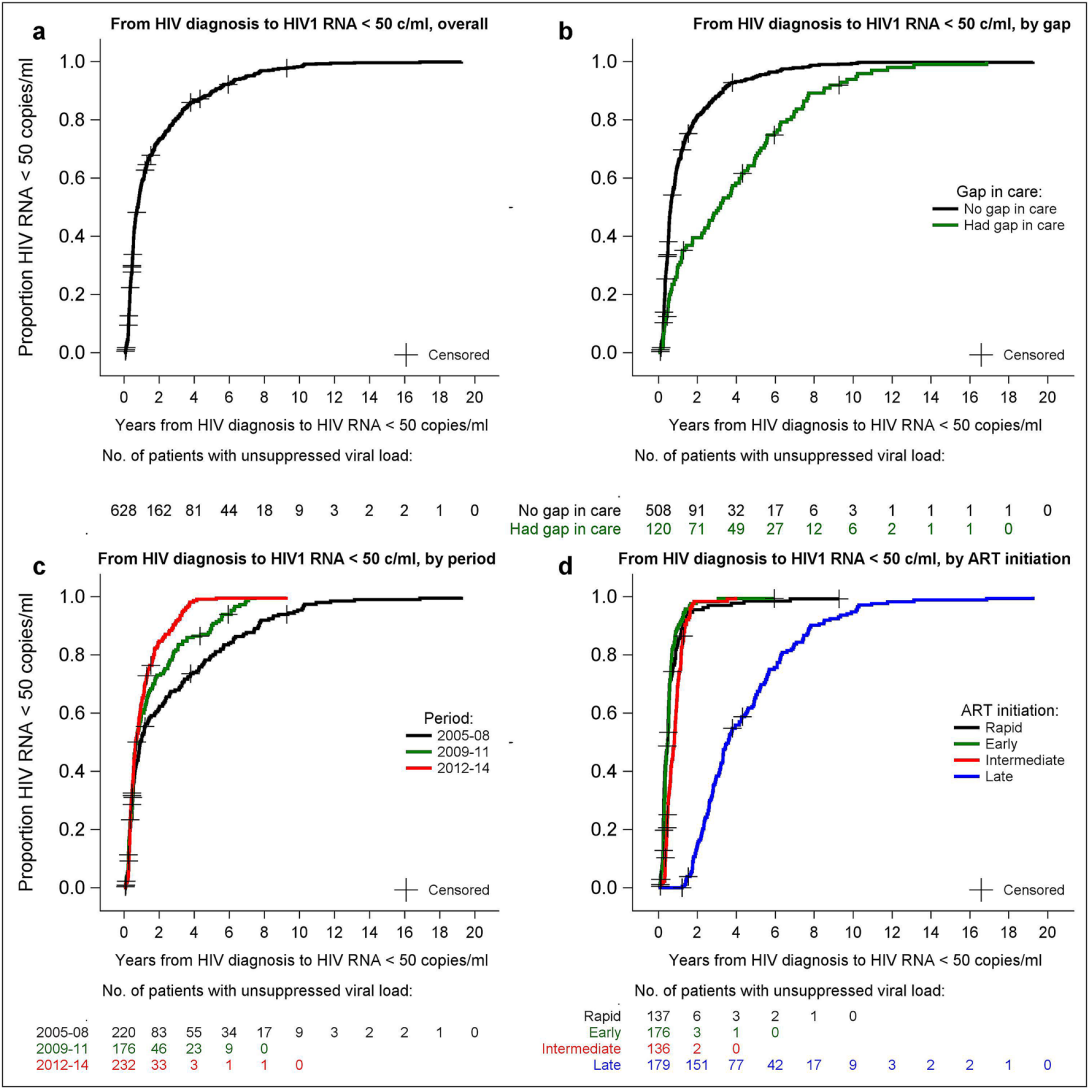
Kaplan–Meier plots of time from HIV diagnosis to undetectable plasma HIV-RNA: (**a**) overall; (**b**) according to gap in care; (**c**) by period of ART initiation; and (**d**) timeliness of ART initiation (rapid = within 7 days early = 8 to 30 days, intermediate = 31 days to one year, late > 1 year).

**Figure 3:**
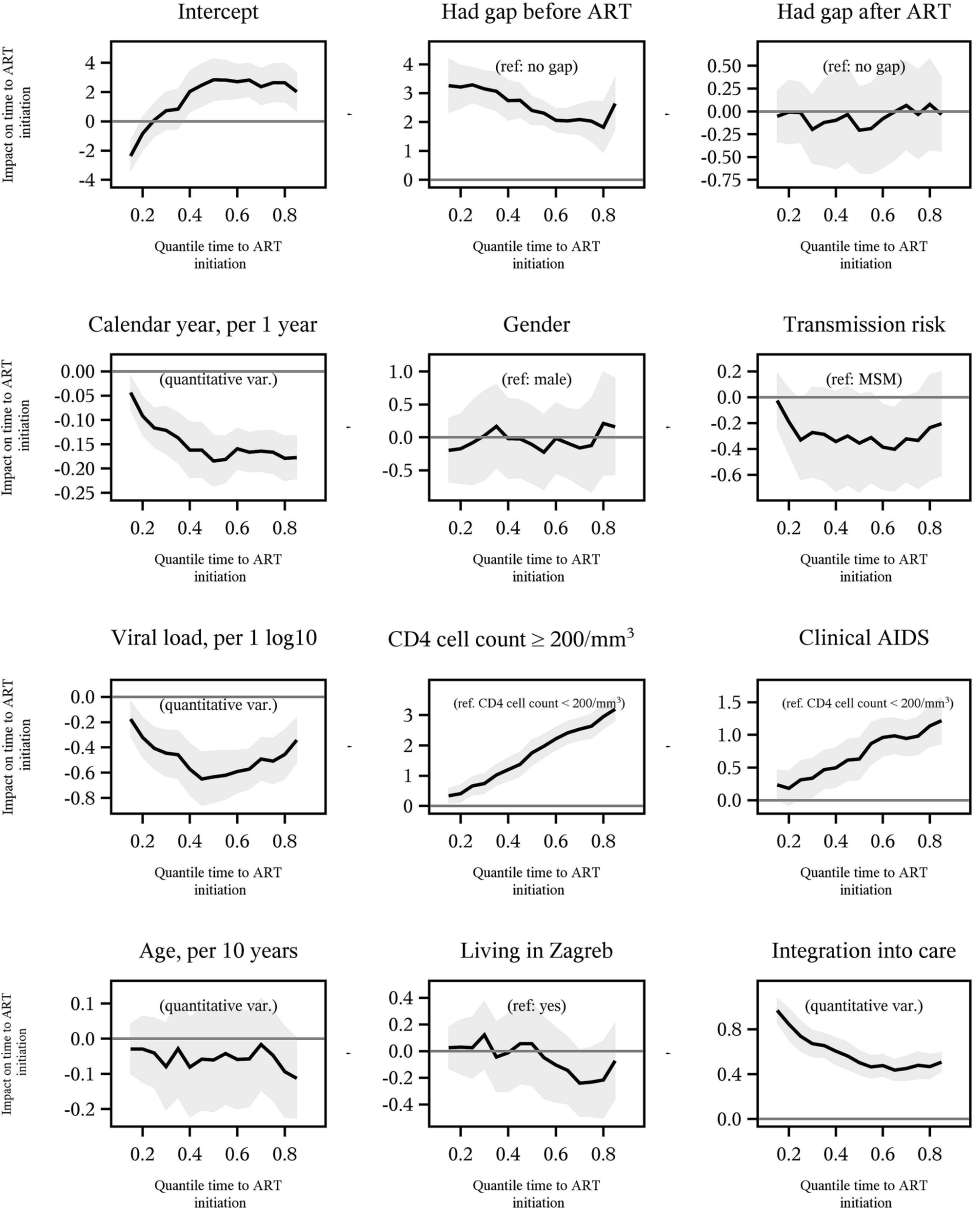
Multivariable quantile regression exploring time from ART initiation and potential related factors. The x-axis represents quantiles of the distribution of the time to ART initiation (log-time in months), and the y-axis represents the parameter estimates (coefficients) change in time associated with a one-unit change of the studied covariate, holding other covariates constant. This change is considered significant at a particular quantile when the associated 95% confidence interval (shaded area) does not cross the 0 line. Integration into care was modeled as log-days.

We estimated the time from HIV diagnosis to reaching undetectable (< 50 copies/mL) HIV-1 RNA by the Kaplan–Meier product-limit survival estimates. Overall, of 628 patients 608 (96.8%) became undetectable during follow-up. Of the 20 who did not, 15 died and 5 were lost to follow-up (LTFU). The median time to reach an undetectable viral load (VL) was 9.6 months (95% CI 7.9–10.6) for the whole study population (Fig. 2a). The median number of months for reaching an undetectable VL decreased by 27.7% from the period 2005–2008 compared to the period 2012–2014 (p < 0.001) (Table 2). Patients who had a gap in care had a longer time to virologic suppression (median 38.2, 95% CI: 26.9–49.9 months) compared to those who did not have a gap (median 7.5, 95% CI 6.8–8.7 months; P < 0.001) (Fig. 2b) (Table 2). Those with a gap in care before ART initiation were suppressed after a median of 67.0 (95% CI 58.9–79.3) months after HIV diagnosis; those who had a gap after ART initiation were suppressed after a median 11.4 (95% CI 6.7–14.1) months. Other significant associations with viral suppression were related to the study period (Fig. 3c), timing of ART initiation (Fig. 3d) and the severity of HIV disease (lower CD4 cell count, higher viral load and clinical AIDS) (Table 2).

Virological failure, defined as one or more plasma HIV-1 RNA measurement > 1000 copies/ml, was observed in 92 (15.3%) of 601 patients who had a viral load test done 6-months after ART initiation (Table 3). Of 51 persons in whom at least one HIV-1 resistance test was done, resistance was detected in 28 (54.9%). Of 131 rapid ART initiators 12 (9.2%) had one or more clinically relevant resistance mutations and this proportion was higher than in the early (4/168, 2.4%), intermediate (5/131, 3.8%) or late (7/171, 4.1%) ART initiation groups. The majority of persons with virological failures were resuppressed to < 50 copies/ml at last HIV-1 RNA measurement (Table 3).

**Table 3:** Number of patients with viral load suppression, failure, resistance and durability of viral suppression according to timeliness of ART initiation, 2005–2014.

	ART initiation after confirmed HIV diagnosis			
	Rapid (within 7 days)	Early 8 to 30 days	Intermediate 31 days–1 year	Late > 1 year
Number of patients with > 6 months follow-up	N = 131	N = 168	N = 131	N = 171
Virologic failure^a^, %, (n)	22.1 (29)	10.1 (17)	16.0 (21)	14.6 (25)
Resistance tests performed at failure, n	17	10	11	13
Any resistance at failure, n	12	4	5	7
Resuppressed^b^ < 50 copies/ml, n/N	23/29	11/17	16/21	16/25
**Durability of viral load suppression**				
Among patients who became undetectable	N = 134	N = 167	N = 132	N = 175
HIV1-RNA < 50 copies/ml^b^, %, (n)	92.5 (124)	97.0 (162)	94.7 (125)	92.6 (162)
HIV1-RNA < 200 copies/ml^b^, %, (n)	94.8 (127)	97.6 (163)	97.7 (129)	94.9 (166)
Time^c^, median (Q1-Q3), years	6.2 (3.8–9.2)	5.2 (3.6–8.0)	6.0 (3.9–8.9)	3.9 (1.9–6.7)
All patients	N = 137	N = 176	N = 136	N = 179
HIV1-RNA < 50 copies/ml^b^, %, (n)	90.5 (124)	92.0 (162)	91.9 (125)	91.1 (163)
HIV1-RNA < 200 copies/ml^b^, %, (n)	92.7 (127)	93.8 (165)	95.6 (130)	93.3 (167)
Time^c^, median (Q1-Q3), years	6.0 (3.8–9.1)	5.1 (3.5–7.8)	5.9 (3.8–8.7)	3.9 (1.9–6.6)

*ART* antiretroviral therapy.

^a^A single viral load measurement of HIV1-RNA > 1000 copies/ml at any time after 6 months of ART initiation (including patients who stopped ART).

^b^Viral load at last measurement.

^c^Time from antiretroviral therapy initiation to last HIV viral load measurement.

Of 608 patients who reached an undetectable viral load 573 (94.2%) had < 50 copies of HIV-1 RNA per ml at the last viral load measurement (median: 5.2, Q1–Q3: 3.3–8.0 years of follow-up). Of the whole study population (N = 628), 574 (91.4%) had < 50 copies of HIV-1 RNA per ml at the last viral load measurement after a median of 5.1 (Q1–Q3: 3.2–7.9) years of follow-up.

### Gap in care

In our cohort, 120 patients (19.1%) experienced at least 1 gap of > 12 months in care, 28 (4.5%) had ≥ 2 gaps and 9 (1.4%) had ≥ 3 gaps in care over their follow-up period. After the first gap 85.0% (102/120) patient had a subsequent visit. After the last gap, altogether 26 of 120 (21.7%) patients had no subsequent visit within 12 months till the end of follow-up and were considered LTFU. Of the 120 persons with a gap in care, 62 (51.7%) had a gap after and 49 (40.8%) before ART start; nine (7.5%) had both a gap before and after ART start.

There was a total of 159 episodes of gaps with a median length of 579 days (Q1–Q3: 415.0–1099.0 days). There were 76 episodes (11.7 per 100 patient years of follow-up) of gap before ART initiation and 83 (2.2 per 100 patient years of follow-up) after ART initiation. The rate ratio suggests that a gap before ART initiation was 5.2 times more likely than a gap after ART initiation (95% CI 3.8–7.1).

### Multivariable analysis

We examined factors related to the time from confirmed HIV diagnosis to ART initiation by quantile regression analysis (Fig. 3). A gap in care before ART initiation, calendar year of entry into care, and markers of more advanced HIV disease (higher viral load, lower CD4 cell count and clinical AIDS) were significantly associated with time of ART initiation at all quantiles. Gap after ART, gender, transmission risk (MSM *vs* not MSM), age and place of residence (Zagreb *vs* outside of Zagreb) did not have a significant impact on time of ART initiation. MSM had a somewhat longer time to ART initiation than non-MSM but this was significant only at the 60th and 65th quantile (p = 0.019 and 0.014 respectively; Fig. 3). As expected, persons who had a gap in care before ART, initiated ART later compared to those with no gap and this difference was decreasing from lower to higher quantiles. The back-transformation of our log-transformed dependent variable indicated that persons with a gap before ART started ART 10.0 (95% CI 6.5–15.7) times later compared to those who did not have a gap at the 50th quantile. Calendar year of entry into care (per one year) was significantly associated with earlier ART initiation. At the 50th quantile, an increase of one year resulted in 17.8% (95% CI: 13.1–22.2) earlier ART initiation. The effect of calendar year on early ART initiation was less pronounced at lower quantiles.

Higher viral load also resulted in earlier ART initiation. At the 50th quantile an increase of one unit (log10) of viral load resulted in 46.5% (95% CI 33.2–57.2) earlier ART initiation. Among persons with no clinical AIDS, those with CD4 count ≥ 200/mm^3^ started ART later in comparison to persons with CD4 count < 200/mm^3^. This difference was more pronounced at higher quantiles, at the 75th quantile, persons with CD4 count ≥ 200/mm^3^ started ART 13.3 (95% CI 8.9–19.9) times later than those with < 200 CD4 cells per mm^3^. Also, persons with clinical AIDS started ART later in comparison to persons with CD4 count < 200/mm^3^, with an increasing difference from low to high quantiles, at the 75th quantile, ART was initiated 2.6 (95% CI 1.8–3.5) times later. The analysis was also adjusted for the time to integration into care, and as expected integration into care was associated with later ART initiation at all quantiles. The effect of integration into care was more pronounced at lower quantiles.

## Discussion

We report on Croatian national data on time from HIV diagnosis to ART initiation for the period 2005 to 2014. The time from diagnosis to ART initiation decreased by 77.8% through the study periods and the overall median time from diagnosis to ART initiation was 31 days. The time from HIV diagnosis to first clinical visit also decreased and it was the shortest in 2012–2014 with a median of 7 days. There are very few national estimates on time from HIV diagnosis to ART initiation. In a population-based probability sample of HIV-infected adults receiving medical care in the United States (May 2004–April 2009) the median time to ART initiation was 10 months. A recent report from England available at a preprint server (arXiv:2010.00740) showed that the percentage of MSM on ART within 180 days of diagnosis increased from 34.7% in 2008 to 84.5% in 2016 and 91.0% in 2018. The North American AIDS Cohort Collaboration on Research and Design study group recently reported that the median days from entry into care to ART prescription was 69 days in 2005, 29 days in 2012, and 6 days in 2018. In 2018, 35% of participants entering HIV care were prescribed ART the same day^14^. Many reports from cohorts, clinic-based or program-based sites have published trends of shorter times to ART initiation in more recent years^13,15–22^ particularly after 2015 when “treat all” policy was introduced by WHO^6^.

Our data indicate that immediate ART provision in Croatia seemed to predate the WHO guideline changes made in 2015. ART initiation within 30 days of HIV diagnosis was already present in almost 30% of persons entering care with a CD4 cell count > 350 per mm^3^ in the period 2012–2014. There is a paucity of data on the durability of viral suppression in early ART starters, our data indicate that early ART initiation is associated with durable viral suppression over a median of 5-year follow-up. Overall, 15% of patients had at least one measurement of HIV-1 RNA > 1000 copies/ml after 6-months of ART, and this figure was highest among rapid ART initiators (29/131, 22%, Table 3). Also, numerically, rapid starters had more HIV-1 resistance at failure (Table 3). However, this should be interpreted with caution as the number of patients in our study was relatively small. It is encouraging that the majority of persons who experienced virological failure where latter resuppressed (Table 3). Nevertheless, more studies on the durability, virological failure and resistance after rapid and early ART initiation are needed.

Differences in the timing of ART initiation among various patient categories may be explained by different factors. For example, a preference for beginning drug therapy as soon as possible for patients with difficulty reaching the treatment center may explain the difference in care among patients who live outside the city versus in Zagreb. Introduction of ART also depended on the severity of HIV infection and thus patients with lower CD4 cell counts and higher viral RNA load initiated ART earlier. Patients with advanced age may have been deemed at risk for poor health outcomes due to HIV and were initiated with ART sooner than younger (possibly deemed healthier) counterparts. Heterosexual mode of transmission is associated with more advanced HIV disease at diagnosis in Croatia^23^, so the longer time to ART initiation in MSM seen on bivariable analysis was largely attenuated in multivariable analysis. However, other factors such as for example fear of side effects might be more pronounced in MSM comparted to heterosexuals which then might have resulted in a delay in ART initiation.

The overall linkage to care in our study was within 7 days from HIV diagnosis in 54.0% and within 30 days in 86.0%, which is higher than reported in some other studies^20,24,25^. Still, the question on how quickly ART should be initiated after the confirmed or even suspected HIV diagnosis has been of major focus in recent years. Several randomized trials showed that rapid ART (including the same day of diagnosis) could improve patient outcomes by reducing loss to care and more rapidly achieving an undetectable viral load and thus reducing the possibility of onward HIV transmission^26–33^. Rapid ART initiation could also decrease health costs^34^. On the other hand, a study on the Dat'AIDS cohort showed that a longer period of time between first clinical visit and ART initiation was associated with a better 1-year retention in care^13^. In our study, the gap in care after early ART initiation was as frequent as in those starting ART later.

We used quantile regression in our analysis which is not frequently used to describe associations. The results of quantile regression are interpreted in a similar way to ordinary least squares regression, however unlike predicting the mean, quantile regression is performed at different quantiles of the dependent variable. So, this method provides a more detailed analysis of associations between variables and could be used in studies examining the timeliness of ART initiation.

The inability to establish causation is a limitation of our observational study. For example, while a delay in ART initiation might cause a gap in care, this delay might also be just a consequence of the gap caused by some other factor. Nevertheless, it is encouraging that early ART initiation, without extensive counseling did not result in worse retention in care and did result in achieving a more rapid viral load suppression. While early ART initiation was initially motivated by having a subsequent visit for patients not living in Zagreb, it has been slowly adopted for all patients. Another concern is measuring time to viral suppression which is dependent on the frequency of viral load measurement, so the true time of becoming undetectable is unknown. For example, in our study the time to ART initiation decreased over time by 77.8% whereas the time to reaching an undetectable viral load decreased by 27.7%. We presented our data on durability of VL by the date of last measurement and not by a certain number of years after ART initiation. This has been done because patients might have regular ART refills at the hospital pharmacy and no viral load measurement. However, since the median time of last viral load measurement from ART initiation was quite long for rapid ART starters (> 6.0 years), we believe that we did provide data suggesting durability of VL suppression in those who start ART rapidly. Virologic failure was defined by a single plasma HIV-1 RNA > 1000 copies/ml which is more stringent that the current WHO definition^7^ which requires two measurements. Confirmation was not required for the definition of virologic failure because of less frequent viral load monitoring, and this is also why did not use other definitions of virological failure (for example confirmed > 50 or > 200 HIV-1 RNA copies/ml). Nevertheless, as the majority of patients who failed were resuppressed to < 50 copies/ml the choice of virologic failure cut-off seems not to be of critical importance for our analysis.

In conclusion, our study adds to the growing data of feasibility and beneficence of early initiation of ART and questions the need of extensive counseling prior to ART initiation. Our data support current HIV care guidelines emphasizing early ART initiation. Earlier initiation of ART was not associated with a more frequent subsequent gap in care and our findings also suggest that early ART initiation may have favorable long term virologic outcomes.

## Methods

### Setting

Croatia has a centralized system of HIV care, and all Croatian citizens have universal health insurance. All PLWH are treated at the University Hospital for Infectious Diseases (UHID) in Zagreb where they receive regular clinical medical evaluations, including measurements of CD4 cell counts and HIV-RNA viral load^35,36^. HIV resistance testing is also available but not regularly performed unless in the event of drug failure. The UHID pharmacy dispenses ART drugs for all PLWH and clinicians verify that the patient has the drugs before he or she leaves the hospital. Since 1997, patient information has been kept in an electronic database which catalogues demographic data, dates of clinic visits, prescriptions of ART drugs, CD4 counts, viral load measurements, and other biochemistry tests.

For patients living outside of Zagreb, the centralized care system means that they must travel to the city for receiving HIV care and treatment and for many the journey may be lengthy or difficult. This led UHID physicians to adopt a policy of reducing appointment frequency when feasible^35^. This means that many patients at the UHID receive ART prescriptions for longer periods of time, usually six months. In a typical case, patients begin ART with a month-long prescription, then the next prescription is for two months and then for three. Three-month long ART prescriptions last until the physician is comfortable with providing the patient ART drugs for six months^35^. This is common when the HIV infection is well controlled and the patient requires clinical examination once or twice per year.

### Study population and definitions

**Figure 4:**
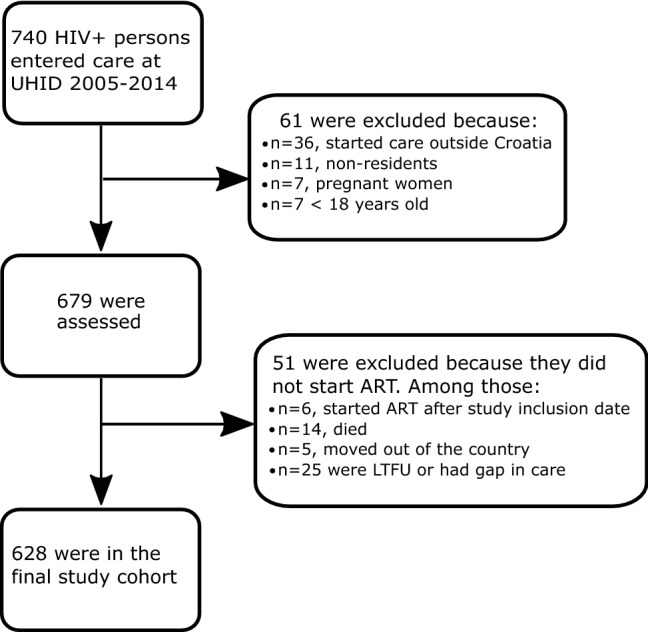
Flow diagram of participants included into the study. *HIV* human immunodeficiency virus; *UHID* University Hospital for Infectious Diseases, Zagreb, Croatia; *ART* antiretroviral treatment; *LTFU* lost to follow-up.

We utilized anonymized data from the electronic UHID database. The cohort was comprised of Croatian citizens or permanent residents at least 18 years of age who arrived to the UHID for HIV treatment between 2005 and 2014 and had not yet received HIV care elsewhere. Pregnant women were excluded from the study. A total of 628 persons who initiated ART were included in the study cohort. The flow diagram for selecting the study cohort is shown in Fig. 4.

The date of HIV diagnosis was defined as the date when blood was drawn for confirmatory testing. The date of a patient’s first clinical visit was considered the starting date of care when a CD4 cell count and viral load was measured. The follow-up ended on December 31, 2017. Patients were censored at the date of their death, relocation from Croatia, or their LTFU. They were considered to have a gap in care if more than one year passed without follow-up. Patients with a gap in care were also considered LTFU if they were alive and living in Croatia and had no outpatient visit for more than 12 months without returning by December 31, 2017.

The study was approved by the Ethics Committee of the University Hospital for Infectious Diseases (UHID), Zagreb, Croatia. The study was performed in accordance with the Declaration of Helsinki and Code of Medical Ethics and Deontology of the Croatian Medical Chamber. Written informed consents was obtained from all participants included in this study.

### Viral load and resistance

HIV viral load was determined by various methods: the Cobas AmpliPrep Cobas Amlicor HIV-1 Monitor Test, Version 1 (lower detection limit 200 copies/ml), the ultrasensitive Amplicor HIV-1 Monitor test (lower detection limit 50 copies/ml), the Cobas Ampliprep/Cobas Taqman HIV-1 test, v2.0 (detection limit 20 copies/ml), all from Roche Molecular Systems, Branchburg, NJ and the Abbott RealTime HIV-1 Viral Load assay (detection limit 40 copies/ml). The Cobas AmpliPrep Cobas Amplicor HIV-1 Monitor Test, Version 1 and the ultrasensitive Amplicor HIV-1 Monitor test were used till the end of 2015. The Cobas Ampliprep/Cobas Taqman HIV-1 test was used from September 2009 till the end of study (December 2017) and the Abbott RealTime HIV-1 Viral Load assay from November 2011 till end of study.

HIV resistance testing was done with HIV-1 sequencing using the TRUGENE HIV-1 Genotyping Kit (Visible Genetics, Toronto, Canada) or by Sanger sequencing as previously described^37,38^. Virologic failure was defined as a single HIV-1 RNA of > 1000 copies/ml at any time after 6 months of ART. Clinically relevant resistance to NRTIs, NNRTIs or PIs was evaluated with Stanford University HIV Drug Resistance Database, Genotypic Resistance Interpretation Algorithm and IAS Drug Resistance Mutation list. A patient was considered to have virological failure with resistance if he harbored at least one mutation from the abovementioned algorithms.

### Statistical methods

The time from HIV diagnosis to ART initiation was categorized: within 7 days (“rapid”), from 8 to 30 days (“early”), from 31 days to one year (“intermediate”), and more than one year (“late”). We assessed how ART initiation timing periods were related to the following: gap in care, gender, transmission mode, CD4 count (cells per µL), HIV-RNA viral load (categorized as greater or less than 10^5^ copies/mL), residence in or outside of Zagreb, whether or not clinical AIDS was present, presence of hepatitis B antigen or hepatitis C antibody positivity at inclusion into care, initial ART type, and year of entry into care. We also assessed the durability of viral load suppression by the proportion of persons with plasma HIV1-RNA < 50 and < 200 copies/ml at last follow-up measurement.

We present our data on the study population by frequencies and percentages or median with first and third quartiles (Q1–Q3). Trends in the timeliness of ART initiation were examined by the Cochran-Armitage test. The Jonckheere-Terpstra test was used for comparison of doubly ordered variables, and the Wilcoxon-Mann–Whitney test was used for comparison of groups with continuous or ordinal variables. The incidence of gap in care before and after ART initiation was assessed by the Poisson method. Kaplan–Meier estimators were used to measure time from HIV diagnosis to antiretroviral treatment initiation and to viral suppression defined as a viral load < 50 copies/mL. Patients were censored if death (n = 15) or lost to follow-up (n = 5) occurred before reaching an undetectable viral load.

We also conducted quantile regression analysis to study factors related to ART initiation with gap in care considered the major independent (predictor) variable. Gap in care was categorized as no gap, gap before ART, and gap after ART start. Persons with both gap before and after ART start were excluded (n = 9), so this analysis included 619 participants. The time from HIV diagnosis to ART initiation was the outcome variable (log months). Age, viral load (log10) calendar year and time to integration into care (log days) were modeled as continuous predictor variables. There were concerns about collinearity between the CD4 cell count and clinical AIDS, so we categorized those variables into three categories: (1) CD4 cell count < 200 per mm^3^ and no clinical AIDS, (2) clinical AIDS regardless of CD4 count and (3) CD4 cell count ≥ 200 per mm^3^ and no clinical AIDS. Other variables included into the model were gender, residence (living in Zagreb *vs* outside of Zagreb), and transmission risk (MSM *vs* not MSM).

Quantile regression has the advantage to provide a more exact description of the distribution of the determinants of time to ART initiation at different quantile (percentile) levels. The coefficients were estimated for each 5 percentiles from 5 to 95th quantile of time to ART initiation. Standard errors and 95% confidence intervals (CI) were estimated using resampling with 1000 repetitions. The coefficients for each quantile were plotted as well as their 95% CI. We used SAS software system release 9.4 (SAS Institute, Cary, NC) to perform the analyses. Two-sided P-values < 0.05 were considered significant.
